# Association of Polycystic Ovary Syndrome with Clinical, Physical, and Reproductive Factors: A Data-Driven Analysis

**DOI:** 10.3390/diagnostics15060711

**Published:** 2025-03-12

**Authors:** Ismat Ara Begum, A. S. M. Sanwar Hosen, Deepak Ghimire, Mi Jin Park

**Affiliations:** 1 Department of Biomedical Sciences and Institute for Medical Science, Jeonbuk National University Medical School, Jeonju 54907, Republic of Korea; ismatara1986@gmail.com; 2 Department of Artificial Intelligence and Big Data, Woosong University, Daejeon 34606, Republic of Korea; 3 IT Application Research Center, Korea Electronics Technology Institute, Jeonju 54853, Republic of Korea; deepak@keti.re.kr; 4 Department of Psychiatry, Seoul St Mary’s Hospital, College of Medicine, The Catholic University of Korea, Seoul 03083, Republic of Korea

**Keywords:** polycystic ovary syndrome (PCOS), anti-Mullerian hormone (AMH), ovarian morphology, hormonal imbalance, follicle count, cycle length

## Abstract

**Background/Objectives**: Polycystic Ovary Syndrome (PCOS) is a multifactorial endocrine disorder with significant clinical and reproductive implications. Identifying dose–response relationships between clinical, physical, and reproductive factors and PCOS can enhance diagnostic accuracy and inform treatment strategies. This study utilized a data-driven approach to analyze the associations between key factors, including age, weight, menstrual cycle length, Anti-Mullerian Hormone (AMH) levels, and follicle count, with PCOS prevalence. **Methods:** A retrospective analysis was conducted on a dataset of 539 participants to determine the optimal ranges of these factors associated with an increased likelihood of PCOS diagnosis. Statistical analyses were conducted using Python, including correlation matrix, univariate and multivariate logistic regression, and dose–response evaluations. **Results:** Our findings demonstrated that the risk of PCOS increases positively in women under 32 years of age. AMH levels above 4.18 ng/mL were strongly associated with PCOS, suggesting that higher AMH levels may reflect excessive follicular activity rather than enhanced ovarian function. Weight was positively correlated with PCOS, emphasizing the role of metabolic disturbances in its pathophysiology. Additionally, menstrual cycle length exhibited a non-linear association with PCOS, with both shortened and prolonged cycles being indicative of hormonal dysregulation. A higher follicle count was consistently linked to PCOS, reinforcing its diagnostic significance. **Conclusions:** This study provides evidence of non-linear dose–response relationships between PCOS and clinical, physical, and reproductive factors. The proposed optimal ranges may serve as valuable reference points for clinicians, aiding in early diagnosis and personalized management strategies for women with PCOS.

## 1. Introduction

Polycystic Ovary Syndrome (PCOS) is a complex endocrine disorder that affects 15–20% of women of reproductive age, with its prevalence varying based on demographic and diagnostic criteria [1,2]. It is characterized by a spectrum of symptoms, including hyperandrogenism, ovulatory dysfunction, and polycystic ovarian morphology [3,4]. As a leading cause of infertility, PCOS has significant physical and psychological impacts. Obesity is frequently associated with PCOS, with rates ranging from 30% to 60%, while infertility affects approximately 50% of these individuals [5]. Despite its widespread impact, the exact pathophysiology of PCOS remains unclear, with genetic, metabolic, and environmental factors contributing to its development. 

Early diagnosis of PCOS is crucial for timely intervention, which can mitigate disease progression and reduce associated comorbidities such as infertility, metabolic disorders, and cardiovascular risks. Delayed diagnosis often leads to prolonged health complications, underscoring the need to identify reliable diagnostic markers and understand the dose–response relationships between clinical and physical factors. Understanding these associations enables earlier management and treatment, ultimately improving patient outcomes [6,7,8].

PCOS is linked to a wide range of health issues, including irregular menstrual cy-cles, infertility, insulin resistance, diabetes, cardiovascular risks, and mental health challenges such as depression and anxiety [9,10,11,12,13,14]. Among these, insulin resistance and hyperinsulinemia play significant roles in the clinical manifestation and progression of PCOS [15]. These metabolic disturbances exacerbate symptoms and increase the risk of comorbidities such as type 2 diabetes and cardiovascular disease [16,17]. The clinical and metabolic heterogeneity of PCOS underscores the importance of exploring dose–response relationships to better understand how varying levels of factors influence the severity and outcomes of the disorder.

Key factors, including age, weight, menstrual cycle length, Anti-Mullerian Hormone (AMH) levels, and follicle number, are thought to significantly influence PCOS severity and patient outcomes. For example, obesity and hirsutism are associated with lower quality-of-life scores, as measured by the modified Ferriman–Gallwey (mFG) scale, which assesses androgen-sensitive areas [18,19,20]. While AMH levels above 3.8–5 ng/mL are increasingly being considered diagnostic markers for PCOS, there is limited consensus on definitive cutoff values. Integrating AMH levels with the Rotterdam criteria has been proposed as a means to improve diagnostic accuracy [21,22,23]. However, much of the existing research treats these factors as categorical variables, limiting the understanding of their continuous, dose-dependent effects.

This study employs a data-driven approach to investigate the dose–response rela-tionships between key clinical, physical, and reproductive factors such as age, weight, menstrual cycle length, AMH levels, and follicle number, in PCOS. By analyzing clinical data, we aim to identify thresholds and ranges at which these factors significantly impact PCOS pathogenesis and patient outcomes. This approach not only enhances our understanding of PCOS but also provides evidence-based guidance for clinicians, paving the way for improved diagnostic precision, personalized management strategies, and earlier interventions that may reduce long-term health risks.

## 2. Methods

### 2.1. Survey Description

The dataset used in this study was obtained from Kaggle, a popular online platform for data science and machine learning. Kaggle provides a wide array of datasets contributed by various users and organizations, allowing for diverse research opportunities. The PCOS dataset, specifically, was collected and shared by researchers to facilitate studies related to PCOS. The dataset includes a comprehensive collection of variables relevant to PCOS, ensuring detailed analysis and insights into the condition. Ethical considerations for data use were followed, with the dataset being made publicly available for research and educational purposes. Users accessing the dataset from Kaggle are expected to adhere to the platform’s guidelines and ethical standards for data usage. This study involves a secondary analysis of an existing dataset related to PCOS. The dataset includes clinical, physical, and reproductive parameters, which are analyzed to identify potential associations with PCOS.

### 2.2. Study Population

**Figure 1:**
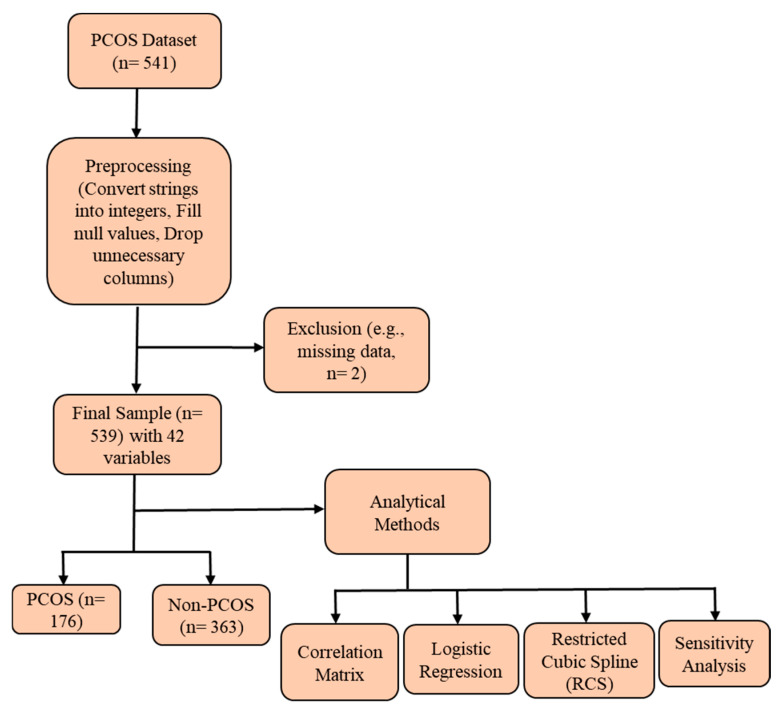
Overview of the study population and analytical methods.

For our study, we utilized the PCOS dataset sourced from Kaggle, which initially included 541 participants. After applying exclusion criteria, such as missing data, 2 participants were excluded, resulting in a final sample size of 539 participants. The dataset comprises 42 variables relevant to the diagnosis and characterization of PCOS. The study population was divided into two groups based on PCOS diagnosis: Confirmed PCOS: 176 participants and Non-PCOS: 363 participants (Figure 1). These groups were analyzed to explore various clinical, physical, and reproductive characteristics associated with PCOS, allowing for a comprehensive examination of the condition and its impacts. The dataset includes information on the number of pregnancies, complications, infertility status, and a variable titled ’Pregnant (%)’, which indicates whether the participant has ever been pregnant at any point in their lifetime, rather than their current pregnancy status. While the dataset provides valuable clinical and reproductive details, it does not include information on the specific geographic region, ethnic background, or clinical setting from which participants were recruited. This is a limitation that should be considered when interpreting the findings, as PCOS prevalence and characteristics may vary across populations.

### 2.3. Statistical Analysis

Data processing, all analyses, and plotting were performed with Python 3.11.7. Continuous numeric variables were expressed as mean ± Standard Deviation (SD), and categorical variables were described as percentages. Student’s *t*-test was used for continuous numeric variables, and the Chi-squared test was used for categorical variables. Multivariate logistic regression analyses were performed to identify the influencing factors of PCOS. The candidate variables for multivariate logistic regression analyses were those with *p* < 0.05 after the univariate analyses. Multivariate logistic regression analyses (using the backward logistic regression method) were conducted by fitting a logistic regression model. Logistic regression results were expressed as Odds Ratios (OR) with 95% Confidence Intervals (CI), and forest plots were generated using Python libraries such as Matplotlib 3.6.3 and Seaborn 0.11.2. The dose–response relationship between variables (Age, Weight, Cycle length, AMH level, Follicle number on the left ovary, and Follicle number on the right ovary) and the OR of PCOS was evaluated using a Restricted Cubic Spline (RCS) with covariates adjusted. Sensitivity analyses were performed to evaluate the stability of our findings by restricting the analytic samples to specific subgroups. For example, analyses were conducted separately for women without regular exercise. A *p*-value of <0.05 was considered statistically significant.

## 3. Results

### 3.1. Baseline Characteristics of the PCOS Group Versus the Non-PCOS Group

The characteristics of the 539 participants in the study are summarized in Table 1. Among these, 176 patients (32.65%) were diagnosed with PCOS, while 363 patients (67.35%) did not have PCOS. Significant differences were observed between the two groups in various clinical, physical, and reproductive factors. Women with PCOS were younger (30.16 ± 5.28 years vs. 32.05 ± 5.36 years, *p* = 0.0001), had a higher BMI (25.51 ± 4.37 vs. 23.74 ± 3.76, *p* = 0.0000), and a shorter cycle length (4.56 ± 1.83 days vs. 5.13 ± 1.27 days, *p* = 0.0002). They also demonstrated higher AMH levels (7.78 ± 7.76 ng/mL vs. 4.54 ± 4.29 ng/mL, *p* = 0.0000) and lower cycle regularity (46.29% vs. 84.57%, *p* = 0.0000). Additionally, PCOS patients had a higher pulse rate (73.83 ± 2.74 bpm vs. 72.96 ± 5.04, *p* = 0.0099) and reported significantly higher incidences of weight gain (68.36% vs. 22.80%, *p* = 0.0000), hair growth (57.06% vs. 12.91%, *p* = 0.0000), and skin darkening (62.15% vs. 15.38%, *p* = 0.0000). In terms of reproductive health, the number of follicles was significantly higher in women with PCOS compared to those without PCOS, both in the left ovary (9.78 ± 4.32 vs. 4.35 ± 2.82, *p* = 0.0000) and the right ovary (10.75 ± 4.17 vs. 4.64 ± 2.93, *p* = 0.0000). Women with PCOS also had larger average follicle sizes in both the left ovary (15.68 ± 2.73 mm vs. 14.68 ± 3.87 mm, *p* = 0.0006) and the right ovary (15.91 ± 3.10 mm vs. 15.22 ± 3.41 mm, *p* = 0.0202). Additionally, the endometrium was thicker in women with PCOS (8.79 ± 1.91 mm vs. 8.32 ± 2.26 mm, *p* = 0.0133). PCOS patients also had a higher waist circumference (34.67 ± 3.76 inches vs. 33.43 ± 3.45 inches, *p* = 0.0003) and hip circumference (38.88 ± 4.17 inches vs. 37.55 ± 3.78 inches, *p* = 0.0004), though the waist-to-hip ratio was not significantly different (*p* = 0.6918). Lifestyle factors also differed between the two groups. Women with PCOS were more likely to consume fast food (78.53% vs. 38.19%, *p* = 0.0000). These findings highlight significant distinctions between the PCOS and non-PCOS groups, particularly in terms of reproductive, metabolic, and lifestyle factors.

### 3.2. Correlation Analysis of Significant Continuous Variables in the Study Population

**Figure 2:**
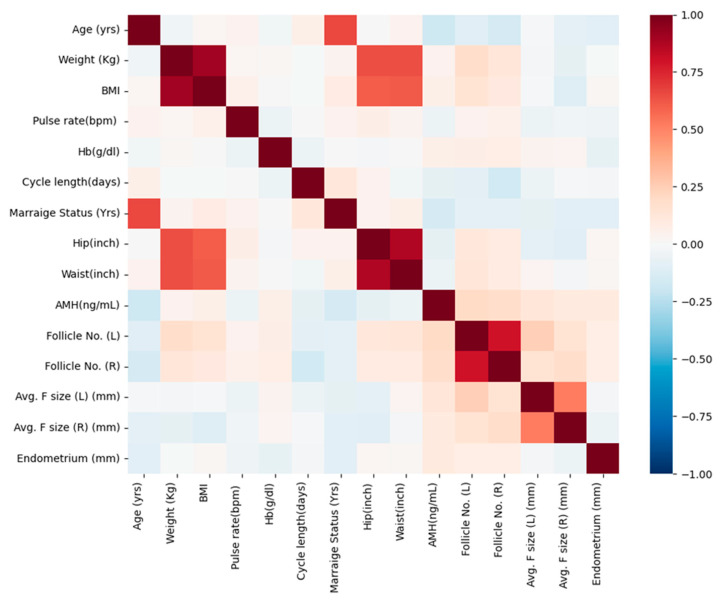
Correlation matrix of significant continuous variables. AMH: Anti-Mullerian Hormone; Follicle No. (L): Follicle No. of Left ovary; Follicle No. (R): Follicle No. of Right ovary.

Correlation analysis was conducted to explore key relationships among significant continuous variables and to assess potential multicollinearity before performing multivariable logistic regression. Our findings reveal several meaningful associations with clinical implications (Figure 2). A strong positive correlation was observed between weight and BMI (r = 0.901), reinforcing the well-established relationship between body weight and obesity-related parameters. Similarly, waist and hip measurements exhibited a strong correlation (r = 0.874), which aligns with their role in assessing body fat distribution, a crucial factor in metabolic health. In the context of ovarian function, the number of follicles in the left and right ovaries showed a strong positive association (r = 0.799), reflecting a consistent pattern of follicular development across both ovaries. While AMH levels demonstrated a weak positive correlation with follicle count (r = 0.200 for Follicle No. (L) and r = 0.183 for Follicle No. (R)), this suggests that AMH may serve as an indicator of ovarian reserve, though its predictive value should be considered alongside other clinical parameters. A weak negative correlation was observed between AMH levels and menstrual cycle length (r = −0.073), hinting at a possible link between ovarian reserve and menstrual cycle regularity. Additionally, endometrial thickness exhibited slight positive correlations with follicle count (Follicle No. (L) (r = 0.076) and Follicle No. (R) (r = 0.075)), which could have implications for endometrial receptivity and overall reproductive potential. By focusing on these relationships, our analysis provides insights into the interplay between body composition, ovarian reserve markers, and reproductive function.

Continuous numeric variables are presented as mean ± Standard Deviation (SD), while categorical variables are expressed as percentages. Student’s *t*-test was used to compare continuous numeric variables, and the Chi-squared test was applied to categorical variables. Blood group values are coded as follows: 11 = A+, 12 = A−, 13 = B+, 14 = B−, 15 = O+, 16 = O−, 17 = AB+, 18 = AB−. Pregnant (%) refers to whether the participant has ever been pregnant, not their current pregnancy status. Abbreviations: BMI: Body Mass Index; RR: Respiratory Rate; Cycle R: Cycle Regular; Cycle I: Cycle Irregular; HCG: Human Chorionic Gonadotropin; FSH: Follicle-Stimulating Hormone; LH: Luteinizing Hormone; TSH: Thyroid-Stimulating Hormone; AMH: Anti-Mullerian Hormone; PRL: Prolactin; PRG: Progesterone; RBS: Random Blood Sugar; Follicle No. (L): Follicle Number of Left ovary; Follicle No. (R): Follicle number of Right ovary.

### 3.3. Association of AMH and Other Factors with PCOS

**Figure 3:**
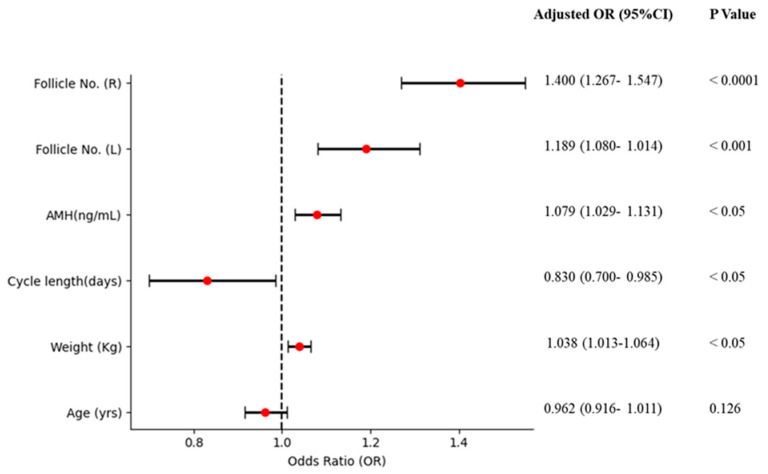
Forest plot of multivariable logistic regression. AMH: Anti-Mullerian Hormone; Follicle No. (L): Follicle No. of Left ovary; Follicle No. (R): Follicle No. of Right ovary. The red circle is adjusted OR.

A multivariate analysis was performed using logistic regression (the backward logistic regression method) to examine the association between PCOS and the candidate variables that had a *p*-value of less than 0.05 after univariate analyses. The candidate variables included age, weight, cycle length, AMH levels, and the number of follicles on the left and right ovaries. In the unadjusted model, age showed a significant association with PCOS, with an OR of 0.934 (95% CI: 0.901–0.968) and a *p*-value of less than 0.001. However, in the adjusted model, age had an OR of 0.962 (95% CI: 0.916–1.011) with a *p*-value of 0.126, indicating no significant association with PCOS. Weight was significantly associated with PCOS in both crude and adjusted analyses. In the crude analysis, the OR was 1.044 (95% CI: 1.026–1.062) with a *p*-value of less than 0.0001, while the adjusted OR was 1.038 (95% CI: 1.013–1.064) with a *p*-value of less than 0.05, showing a significant positive association with PCOS. Cycle length also showed a significant inverse association with PCOS. The crude OR was 0.750 (95% CI: 0.654–0.862) with a *p*-value of less than 0.0001, and the adjusted OR was 0.830 (95% CI: 0.700–0.985) with a *p*-value of less than 0.05, confirming the inverse relationship. AMH levels were strongly associated with PCOS, with a crude OR of 1.108 (95% CI: 1.069–1.149) and a *p*-value of less than 0.0001. After adjustment, the OR remained significant at 1.079 (95% CI: 1.029–1.131) with a *p*-value of less than 0.05, indicating a positive association with PCOS. Follicle numbers in the left ovary showed a strong positive association, with a crude OR of 1.533 (95% CI: 1.422–1.652) and a *p*-value of less than 0.0001. The adjusted OR was 1.189 (95% CI: 1.080–1.014) with a *p*-value of less than 0.001, maintaining significance. Similarly, follicle numbers in the right ovary showed significant association with PCOS, with a crude OR of 1.605 (95% CI: 1.481–1.739) and a *p*-value of less than 0.0001. In the adjusted models, the OR was 1.400 (95% CI: 1.267–1.547) with a *p*-value of less than 0.0001, indicating a significant positive association (Table 2 and Figure 3). In sensitivity analyses, the association of these factors with PCOS was similar to the results observed in women who did not engage in regular exercise (*n* = 406) (Table 2).

**Figure 4:**
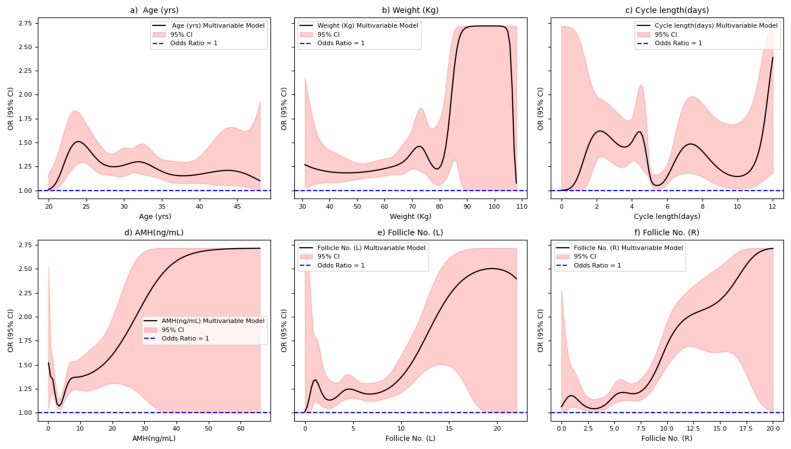
Association of the six factors with PCOS: (**a**) Association of age with PCOS; (**b**) Association of weight with PCOS; (**c**) Association of cycle length with PCOS; (**d**) Association of AMH with PCOS; (**e**) Association of follicle no. (L) with PCOS; (**f**) Association of follicle no. (R) with PCOS. AMH: Anti-Mullerian Hormone; Follicle No. (L): Follicle No. of Left ovary; Follicle No. (R): Follicle No. of Right ovary.

We further evaluated the dose–response relationship between PCOS and various factors, including age, weight, cycle length, AMH levels, and follicle number, using RCS. The results are presented in Figure 4. After adjusting weight, cycle length, AMH levels, follicle no. (L) and follicle no. (R), the likelihood of PCOS increased non-significantly with increasing age (non-linear, *p* = 0.126, Figure 4a). We found that the association between PCOS and age showed a notable increase at 24 years (OR: 1.50, 95% CI: 1.25–1.75). The OR for PCOS was 1.25 (95% CI: approximately 1.01–1.70) at the age of 45 years. After adjusting age, cycle length, AMH levels, follicle no. (L) and follicle no. (R), the likelihood of PCOS increased significantly with increasing weight (non-linear, *p* < 0.05, Figure 4b). We estimated a strong association between weight and PCOS risk, particularly above 80 kg, with an OR of around 2.75 (95% CI: 1.35–2.75). After adjusting age, weight, AMH levels, follicle no. (L) and follicle no. (R), the likelihood of PCOS was associated with cycle length (non-linear, *p* < 0.05, Figure 4c). The OR fluctuates across different cycle lengths, showing an increased risk at both very short and long cycles, with an OR of approximately 1.50 (95% CI: 1.10–2.05) for cycle lengths above 10 days. After adjusting age, weight, cycle length, follicle no. (L) and follicle no. (R), the likelihood of PCOS increased significantly with increasing AMH levels (non-linear, *p* < 0.05, Figure 4d). A rising OR was observed with higher AMH levels, reaching 1.80 (95% CI: 1.20–1.40) at 5 ng/mL. After adjusting age, weight, cycle length, AMH levels, and follicle no. (R), the likelihood of PCOS was positively correlated with the follicle no. (L) (non-linear, *p* < 0.001, Figure 4e). Figure 4e showed an increasing OR with higher follicle counts, with an OR of about 2.25 (95% CI: 1.40–2.55) for 15 follicles. After adjusting age, weight, cycle length, AMH levels, and follicle no. (L), the likelihood of PCOS was positively correlated with the follicle no. (R) (non-linear, *p* < 0.0001, Figure 4f). The highest OR was observed for 20 follicles (OR 2.75, 95% CI: 1–2.75).

In sensitivity analyses, the relationships between PCOS and key variables (age, weight, cycle length, AMH levels, follicle no. (L), and follicle no. (R)) were similar to the findings observed in the entire study population, even when analyzed separately for women without regular exercise (Figure S1). These results suggest that the dose–response associations between PCOS and these factors remain consistent across different subgroups, reinforcing the robustness of our findings.

## 4. Discussion

This study provides valuable insights into the multifaceted nature of PCOS by examining the associations between clinical, physical, and reproductive health factors with the prevalence of the condition. Our analysis revealed non-linear relationships between several variables—including age, AMH levels, weight, cycle length, and follicle count—and their association with PCOS. These findings underscore the complexity of PCOS diagnosis and management, emphasizing the need for a multifactorial approach to clinical assessment.

Our findings indicate a non-linear relationship between age and PCOS risk. While age is often considered a determinant of reproductive health, our analysis showed that the odds ratio (OR) remained relatively stable across age groups, with a noticeable in-crease at the age of 24. This trend suggests that while reproductive aging—characterized by reduced ovarian reserve and decreased oocyte quality—may contribute to PCOS risk in older women, younger women may also experience significant associations. In our study, a positive association with PCOS was observed even in women under the age of 32. This indicates that while age is an important factor, it may not be the sole determinant of PCOS in younger women. Notably, in crude analysis (Table 2), age showed a significant association with PCOS (*p* < 0.001). However, in the adjusted model (Table 2), the association between age and PCOS was not statistically significant (*p* = 0.126). This suggests that other confounding factors—such as weight, menstrual cycle length, AMH levels, and follicle number in both ovaries—may have influenced the relationship between age and PCOS. Once these factors were accounted for in the adjusted model, the independent effect of age on PCOS risk was reduced, making the association non-significant. These findings highlight the need for a comprehensive evaluation of reproductive health that considers not only age but also other reproductive and physiological factors. Our results align with prior and recent studies, which emphasize the interplay between age, ovarian function, and PCOS phenotypes [24,25,26,27].

AMH levels, a key hormonal marker of ovarian reserve, were strongly positively associated with PCOS. In our study, AMH levels higher than 4.18 ng/mL were linked to an increased risk of PCOS. While AMH is often used to predict ovarian reserve, it has become apparent that higher AMH levels may reflect a larger quantity of follicles, which could be indicative of ovarian dysfunction in PCOS [28]. This aligns with findings suggesting that elevated AMH levels may serve as a marker of follicular excess rather than an indicator of ovarian quality [29,30,31]. Interestingly, elevated AMH levels were strongly associated with the presence of PCOS in our study. This supports the idea that higher AMH levels may reflect follicular excess, a hallmark of PCOS [28,32]. However, it is important to note that while AMH is often elevated in women with PCOS, excessively high levels may indicate underlying ovarian dysfunction rather than necessarily enhancing reproductive outcomes [33]. Our findings suggest that while AMH is a useful marker for diagnosing PCOS, there may be diminishing returns with very high AMH levels, as they may be associated with more severe forms of the condition, including increased risk of anovulation and metabolic disturbances [34].

Weight exhibited a significant positive association with PCOS, reinforcing the well-established link between metabolic disturbances and the syndrome. Women in the higher weight category had a stronger association with PCOS, emphasizing the role of obesity in exacerbating hormonal imbalances [35,36]. Recent findings suggest that weight management strategies, including lifestyle interventions, dietary modifications, and exercise, can significantly improve PCOS symptoms by restoring ovulatory function and reducing metabolic risks [37,38]. Notably, in our study, BMI did not retain its significance in the adjusted model due to collinearity, whereas weight remained a robust predictor, indicating its direct impact on PCOS pathophysiology.

Cycle length was another critical variable in this study. In our dataset, cycle length refers to the duration of menstrual bleeding. Women with shorter cycle lengths were more likely to have PCOS, aligning with existing literature that suggests irregular menstrual cycles, including shorter bleeding durations, are a common feature of the syndrome [39,40]. A shortened bleeding duration could be indicative of hormonal dysregulation, which is typical of PCOS [41]. However, our findings also suggest that longer cycle lengths, often associated with anovulation, are not exempt from increasing the risk of PCOS [42,43]. This non-linear relationship between cycle length and PCOS risk highlights the need for a more nuanced understanding of menstrual irregularities and their underlying causes, beyond simply the duration of bleeding.

The number of follicles in both ovaries was strongly associated with the presence of PCOS. Women with PCOS had a significantly higher follicle count, which is consistent with the characteristic polycystic ovaries seen in this syndrome [44]. These findings further support the notion that follicular excess is a hallmark of PCOS and is likely a result of impaired folliculogenesis, which leads to the accumulation of immature follicles [45].

Overall, this study provides important insights into the factors influencing PCOS, including hormonal markers such as AMH, reproductive health parameters like follicle count, and physical factors. The non-linear relationships between these variables emphasize the need for a comprehensive and personalized approach to diagnosing and managing PCOS. While some factors, such as age and weight, showed clear associations with PCOS risk, others, like AMH and follicle count, revealed more complex interactions that could inform future research and clinical practice.

**Strengths and Limitations:** Our study has several strengths, including a diverse sample size and a comprehensive analysis incorporating correlation assessments, multivariate logistic regression, and sensitivity analyses. These methodological approaches enhance the validity of our findings and provide a robust understanding of PCOS risk factors. However, certain limitations must be acknowledged. The retrospective nature of the study and the absence of specific data on several clinically relevant variables in the dataset, including insulin levels, metabolic disorder indicators, and key lifestyle factors such as physical activity, dietary habits, and sleep patterns, limit our ability to explore additional contributors to PCOS risk. Furthermore, the dataset lacks information on geographic location and ethnicity, factors known to influence PCOS presentation. Additionally, the lack of data on oral contraceptive use, a variable that can significantly impact AMH levels, cycle length, and weight, presents a limitation in fully elucidating PCOS risk factors.

Interestingly, our model adjustments revealed that BMI was not a significant predictor of PCOS when controlling for other factors. Instead, weight remained a stronger predictor, suggesting that BMI may not be the most appropriate measure in this context, possibly due to its collinear relationship with other metabolic variables. This finding underscores the importance of refining diagnostic criteria and risk assessment models to improve PCOS identification and management.

**Future Directions:** Future prospective studies with larger, multicenter cohorts are needed to validate these findings and refine diagnostic criteria for PCOS. Additionally, incorporating advanced biomarkers, metabolic indicators, and genetic predispositions may enhance our understanding of PCOS heterogeneity. Investigating the impact of lifestyle interventions and longitudinal changes in weight, AMH levels, and menstrual cycle patterns will provide deeper insights into the progression and management of PCOS.

## 5. Conclusions

This study provides new insights into the complex interplay of clinical, physical, and reproductive factors associated with PCOS using a data-driven approach. Unlike previous studies that primarily categorize risk factors, our analysis highlights the non-linear, dose–response relationships between key variables such as age, AMH levels, weight, cycle length, and follicle count. Notably, our findings suggest that higher AMH levels may serve as an indicator of follicular excess rather than solely ovarian reserve, emphasizing its potential role in PCOS severity assessment. Additionally, our study reinforces that weight, rather than BMI, is a stronger predictor of PCOS, suggesting the need to reconsider metabolic risk assessments in PCOS diagnosis. Furthermore, this study identifies a non-linear relationship between menstrual cycle length and PCOS risk, suggesting that both shorter and longer cycle lengths are linked to hormonal dysregulation and anovulation, warranting a more nuanced approach to menstrual irregularities in PCOS assessment. These findings contribute to refining diagnostic criteria and improving early identification strategies, supporting the need for personalized interventions. While the etiology of PCOS remains multifactorial, this work provides novel insights into how specific clinical and reproductive markers interact beyond traditional categorical classifications. By integrating continuous variable assessments, clinicians can better predict PCOS risk and tailor interventions accordingly.

Future research should focus on validating these findings in larger, multicenter cohorts while incorporating metabolic and genetic markers to further elucidate PCOS heterogeneity. Understanding the dynamic interactions between clinical and hormonal factors will aid in developing more precise diagnostic models and personalized treatment strategies, ultimately improving patient outcomes.

## Figures and Tables

**Table 1:** Baseline characteristics according to the PCOS and non-PCOS group.

Characteristic	Total	PCOS	Non-PCOS	*p* Value
	(*n* = 539)	(*n* = 176)	(*n* = 363)	
Age (yrs)	31.44 ± 5.40	30.16 ± 5.28	32.05 ± 5.36	0.0001
Weight (kg)	59.68 ± 11.02	63.12 ± 12.08	58.01 ± 10.06	0.0000
Height (cm)	156.51 ± 6.04	157.12 ± 6.17	156.21 ± 5.95	0.1071
BMI	24.32 ± 4.05	25.51 ± 4.37	23.74 ± 3.76	0.0000
Blood Group (%)				0.9570
11	20	19.3	20.4	
12	2.4	2.3	2.5	
13	25.0	23.9	25.6	
14	3.0	3.4	2.8	
15	38.2	37.5	38.6	
16	3.5	4.5	3.0	
17	7.8	9.1	7.2	
18	0.4	0.6	0.3	
Pulse rate (bpm)	73.24 ± 4.44	73.83 ± 2.74	72.96 ± 5.04	0.0099
RR (breaths/min)	19.24 ± 1.69	19.34 ± 1.65	19.20 ± 1.71	0.3531
Hb (g/dl)	11.16 ± 0.87	11.27 ± 0.83	11.11 ± 0.88	0.0347
Cycle (%)				0.0000
R	72.12	46.29	84.57	
I	27.88	53.71	15.43	
Cycle length (days)	4.94 ± 1.49	4.56 ± 1.83	5.13 ± 1.27	0.0002
Marriage Status (Yrs)	7.67 ± 4.81	6.89 ± 4.71	8.06 ± 4.82	0.0079
Pregnant (%)				0.6012
Yes	38.22	36.16	39.01	
No	61.78	63.84	60.99	
No. of abortions	0.29 ± 0.69	0.23 ± 0.55	0.32 ± 0.75	0.1446
I beta-HCG (mIU/mL)	666.93 ± 3354.91	535.05 ± 2930.93	730.87 ± 3544.34	0.4981
II beta-HCG (mIU/mL)	239.11 ± 1606.74	269.06 ± 1911.04	224.59 ± 1438.96	0.7847
FSH (mIU/mL)	14.64 ± 217.42	5.18 ± 5.76	19.23 ± 264.91	0.313
LH (mIU/mL)	6.48 ± 86.83	14.45 ± 151.91	2.62 ± 2.10	0.3027
FSH/LH	6.91 ± 60.80	5.34 ± 25.09	7.68 ± 72.03	0.5796
Hip (inch)	37.98 ± 3.96	38.88 ± 4.17	37.55 ± 3.78	0.0004
Waist (inch)	33.83 ± 3.60	34.67 ± 3.76	33.43 ± 3.45	0.0003
Waist/Hip Ratio	0.89 ± 0.05	0.89 ± 0.05	0.89 ± 0.05	0.6918
TSH (mIU/L)	2.96 ± 3.72	2.93 ± 2.83	2.97 ± 4.09	0.8994
AMH (ng/mL)	5.60 ± 5.86	7.78 ± 7.76	4.54 ± 4.29	0.0000
PRL (ng/mL)	24.38 ± 14.96	24.48 ± 13.91	24.33 ± 15.47	0.9067
Vit D3 (ng/mL)	49.99 ± 346.85	92.71 ± 605.64	29.27 ± 12.38	0.1664
PRG (ng/mL)	0.61 ± 3.82	0.37 ± 0.17	0.73 ± 4.65	0.1414
RBS (mg/dl)	99.85 ± 18.59	101.19 ± 23.69	99.20 ± 15.53	0.3122
Weight gain (%)				0.0000
Yes	37.66	68.36	22.80	
No	62.34	31.64	77.20	
Hair growth (%)				0.0000
Yes	27.27	57.06	12.91	
No	72.73	42.94	87.09	
Skin darkening (%)				0.0000
Yes				
No	30.61	62.15	15.38	
	69.39	37.85	84.62	
Hair loss (%)				0.0001
Yes	45.27	57.62	39.29	
No	54.73	42.94	60.71	
Pimples (%)				0.0000
Yes	48.98	69.49	39.01	
No	51.02	30.51	60.99	
Fast food (%)				0.0000
Yes	51.39	78.53	38.19	
No	48.61	21.47	61.81	
Reg. Exercise (%)				0.1718
Yes	24.49	28.81	22.80	
No	75.51	71.19	77.20	
BP_Systolic (mmHg)	114.68 ± 7.37	114.83 ± 5.55	114.61 ± 8.11	0.7083
BP_Diastolic (mmHg)	76.95 ± 5.57	77.27 ± 4.47	76.80 ± 6.03	0.3057
Follicle No. (L)	6.13 ± 4.23	9.78 ± 4.32	4.35 ± 2.82	0.0000
Follicle No. (R)	6.63 ± 4.44	10.75 ± 4.17	4.64 ± 2.93	0.0000
Avg. F size (L) (mm)	15.01 ± 3.57	15.68 ± 2.73	14.68 ± 3.87	0.0006
Avg. F size (R) (mm)	15.45 ± 3.32	15.91 ± 3.10	15.22 ± 3.41	0.0202
Endometrium (mm)	8.47 ± 2.16	8.79 ± 1.91	8.32 ± 2.26	0.0133

**Table 2:** Association of AMH and other factors with PCOS.

	Unadjusted Model		Adjusted Model	
Parameter	Crude OR (95% CI)	*p* Value	Adjusted OR (95% CI)	*p* Value
**Total population (*n* = 541)**				
Age (yrs)	0.934 (0.901–0.968)	<0.001	0.962 (0.916–1.011)	0.126
Weight (kg)	1.044 (1.026–1.062)	<0.0001	1.038 (1.013–1.064)	<0.05
Cycle length (days)	0.750 (0.654–0.862)	<0.0001	0.830 (0.700–0.985)	<0.05
AMH (ng/mL)	1.108 (1.069–1.149)	<0.0001	1.079 (1.029–1.131)	<0.05
Follicle No. (L)	1.533 (1.422–1.652)	<0.0001	1.189 (1.080–1.014)	<0.001
Follicle No. (R)	1.605 (1.481–1.739)	<0.0001	1.400 (1.267–1.547)	<0.0001
**Women Without Regular** **Exercise (*n* = 406)**				
Age (yrs)	0.955 (0.918–0.994)	<0.05	0.993 (0.938–1.051)	0.802
Weight (kg)	1.047 (1.027–1.067)	<0.0001	1.036 (1.009–1.064)	<0.05
Cycle length (days)	0.678 (0.573–0.803)	<0.0001	0.774 (0.631–0.949)	<0.05
AMH (ng/mL)	1.110 (1.065–1.159)	<0.0001	1.060 (1.005–1.119)	<0.05
Follicle No. (L)	1.549 (1.417–1.694)	<0.0001	1.205 (1.075–1.351)	<0.001
Follicle No. (R)	1.589 (1.450–1.740)	<0.0001	1.369 (1.220–1.535)	<0.0001

OR: Odds Ratio; CI: Confidence Interval.

## Data Availability

A publicly available dataset was analyzed in this study. These data can be found at https://www.kaggle.com/ (accessed on 10 December 2024).

## Supplementary Materials

The following supporting information can be downloaded at: https://www.mdpi.com/article/10.3390/diagnostics15060711/s1, Figure S1: Association of the six factors with PCOS in women without regular exercise.
