# Supplementary material for: Association of Polycystic Ovary Syndrome with Clinical, Physical, and Reproductive Factors: A Data-Driven Analysis
Source: Diagnostics (Basel). 2025 Mar 12;15(6):711. doi: 10.3390/diagnostics15060711 (PMC11941334; doi:10.3390/diagnostics15060711)
Supplement: Supplementary file 1 [file diagnostics-15-00711-s001.zip › diagnostics-3493373-supplementary.pdf]

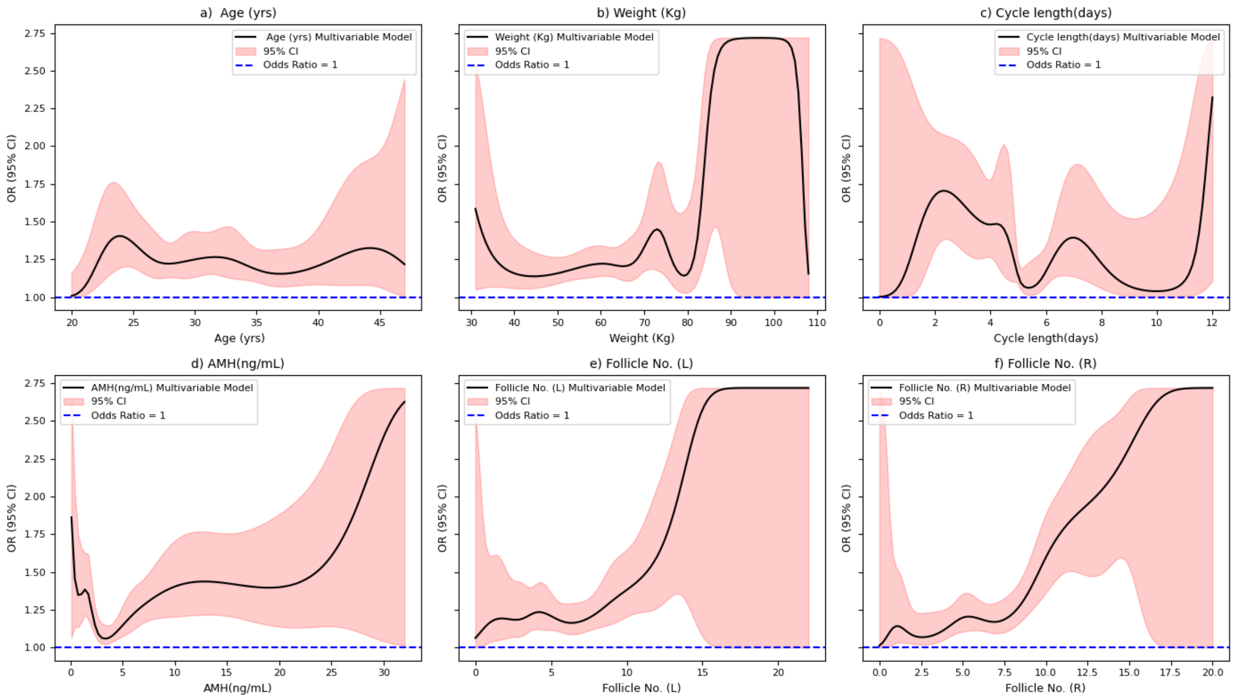

Figure S1: Association of the six factors with PCOS in women without regular exercise ((a). Association of age with PCOS; (b). Association of weight with PCOS; (c). Association of cycle length with PCOS; (d). Association of AMH with PCOS; (e). Association of follicle no. (L) with PCOS; (f) Association of follicle no. (R) with PCOS). AMH: Anti-Mullerian Hormone; Follicle No. (L): Follicle No. of Left ovary; Follicle No. (R): Follicle No. of Right ovary.
